# Supplementary figures and images for: Biodegradation Potential of C7-C10 Perfluorocarboxylic Acids and Data from the Genome of a New Strain of Pseudomonas mosselii 5(3)
Source: Toxics. 2023 Dec 8;11(12):1001. doi: 10.3390/toxics11121001 (PMC10748008; doi:10.3390/toxics11121001)

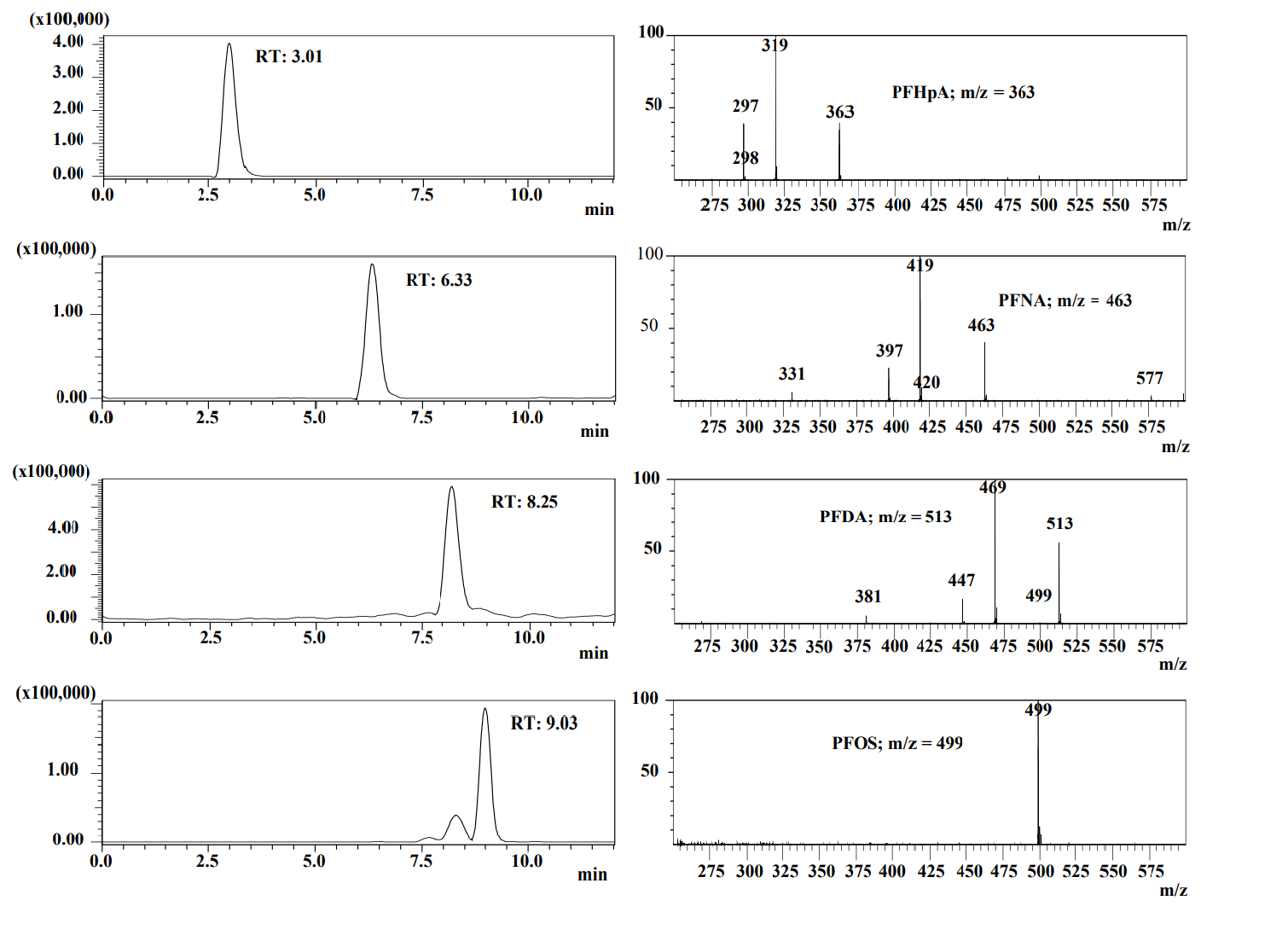

Supplement: Supplementary file 1 [file toxics-11-01001-s001.zip › toxics-2727228-supplementary.jpg]
